# Conversion of epidermal growth factor receptor 2 and hormone receptor expression in breast cancer metastases to the brain

**DOI:** 10.1186/bcr3244

**Published:** 2012-08-16

**Authors:** Renata Duchnowska, Rafał Dziadziuszko, Tomasz Trojanowski, Tomasz Mandat, Waldemar Och, Bogumiła Czartoryska-Arłukowicz, Barbara Radecka, Wojciech Olszewski, Franciszek Szubstarski, Wojciech Kozłowski, Bożena Jarosz, Wojciech Rogowski, Anna Kowalczyk, Janusz Limon, Wojciech Biernat, Jacek Jassem

**Affiliations:** 1 Department of Oncology, Military Institute of Medicine, 128 Szaserów St., 00-909 Warsaw, Poland; 2 Department of Oncology and Radiotherapy, Medical University of Gdańsk, 7 Dębinki St., 80-211 Gdańsk, Poland; 3 Department of Neurosurgery and Children's Neurosurgery Clinic, Medical University of Lublin, 8 Jaczewskiego St., 20-954 Lublin, Poland; 4 Department of Neurosurgery, Maria Skłodowska-Curie Memorial Cancer Center and Institute of Oncology, 5 Roentgena St., 02-781 Warsaw, Poland; 5 Department of Neurosurgery, Voivodal Specialistic Hospital, 18 Żołnierska St., 10- 561 Olsztyn, Poland; 6 Department of Clinical Oncology, Białystok Oncology Center, 12 Ogrodowa St., 15-027 Białystok, Poland; 7 Department of Clinical Oncology, Opole Oncology Center, 66A Katowicka St., 45-060 Opole, Poland; 8 Department of Pathology, Maria Skłodowska-Curie Memorial Cancer Center and Institute of Oncology, 5 Roentgena St., 02-781 Warsaw, Poland; 9 Department of Pathology, Lublin Oncology Center, 7 Jaczewskiego St., 20-090 Lublin, Poland; 10 Department of Pathomorphology, Military Institute of Medicine, 128 Szaserów St., 00-909 Warsaw, Poland; 11 Department of Chemotherapy, Warmia and Masuria Oncology Center, 37 Wojska Polskiego Ave., 10-228 Olsztyn, Poland; 12 Department of Biology and Genetics, Medical University of Gdańsk, 1 Dębinki St., 80-211 Gdańsk, Poland; 13 Department of Pathomorphology, Medical University of Gdańsk, 17 Smoluchowskiego St., 80-214 Gdańsk, Poland

## Abstract

**Introduction:**

We investigated the status of estrogen receptor alpha (ERα), progesterone receptor (PR), and epidermal growth factor receptor 2 (HER2) in primary tumor and in the corresponding brain metastases in a consecutive series of breast cancer patients. Additionally, we studied factors potentially influencing conversion and evaluated its association with survival.

**Methods:**

The study group included 120 breast cancer patients. ERα, PR, and HER2 status in primary tumors and in matched brain metastases was determined centrally by immunohistochemistry and/or fluorescence *in situ *hybridization.

**Results:**

Using the Allred score of ≥ 3 as a threshold, conversion of ERα and PR in brain metastases occurred in 29% of cases for both receptors, mostly from positive to negative. Conversion of HER2 occurred in 14% of patients and was more balanced either way. Time to brain relapse and the use of chemotherapy or trastuzumab did not influence conversion, whereas endocrine therapy induced conversion of ERα (*P *= 0.021) and PR (*P *= 0.001), mainly towards their loss. Receptor conversion had no significant impact on survival.

**Conclusions:**

Receptor conversion, particularly loss of hormone receptors, is a common event in brain metastases from breast cancer, and endocrine therapy may increase its incidence. Receptor conversion does not significantly affect survival.

## Introduction

The brain is a common site of relapse in breast cancer, with an overall occurrence of 10% to 16% in historical series [1]. The highest risk of brain relapse is associated with triple negative and epidermal growth factor receptor 2 (HER2)-positive phenotypes, high tumor grade, and specific molecular tumor signatures [2-4]. Within the last several decades, the incidence of brain metastases in breast cancer patients has been increasing, which has been attributed to several factors, including the use of novel cytotoxic agents [5,6], prolonged survival of patients in an advanced stage, allowing more time for the development of brain relapse, and the impact of targeted systemic therapies [7].

HER2 status as well as expression of hormone receptors (HRs) (that is, estrogen receptor (ER) and progesterone receptor (PR)), are routinely determined in primary breast cancers, and metastatic lesions have typically been assumed to maintain the original phenotype. However, several recent studies indicated a relatively large phenotypic discordance between primary breast cancer and distant metastases. Receptor conversion seems to include mainly loss of HRs [8-13], whereas HER2 alterations are less common [4,8,9,12-16].

Brain metastases from breast cancer have traditionally been managed with surgery or radiotherapy, and the phenotypes of these lesions have received little attention. Even if systemic therapies were used, selection of treatment was usually guided by the phenotype of the primary tumor. However, with the increasing role of targeted systemic therapies, determination of brain metastasis phenotype may have larger therapeutic implications.

Most studies investigating changing phenotypes in recurrent breast cancer have included various locations of metastases, of which brain metastases constituted only a minority. A few studies specifically addressing brain metastases were relatively small or were restricted to selected markers [4,17-22]. In consequence, the knowledge on the conversion rate of HRs and HER2 in brain metastases is limited, and the clinical implications of this phenomenon remain unknown.

Here, we compared the status of ERα, PR, and HER2 in primary tumors and in paired excised brain metastases in a relatively large series of breast cancer patients, assessed the impact of factors potentially influencing receptor conversion, and evaluated association of particular phenotypic changes with survival.

## Materials and methods

### Patients

This multicenter study was approved by the institutional review board of the coordinating center (Medical University of Gdansk, Poland). The archives of neurosurgery and pathology departments were searched to identify eligible patients. We used archival formalin-fixed paraffin-embedded blocks, and all personal data were made anonymous and coded; therefore patient consent was not sought. Inclusion criteria included female sex and diagnosis of unilateral breast cancer with synchronous or metachronous excised brain metastases. All forms of surgical therapy to primary tumor, radiotherapy and systemic therapy, before and after brain relapse, were allowed.

Demographic and clinicopathologic data, as well as clinical follow-up, were extracted from institutional databases or original patient files. The time to brain metastases was calculated from the initial diagnosis of breast cancer to excision of the metastatic lesion. Most brain metastases were symptomatic, but some cases were detected accidentally.

All formalin-fixed paraffin-embedded tissue blocks were centrally collected, and new sections were cut and routinely stained (hematoxylin-eosin, H&E). H&E-stained slides were reviewed by a board-certified pathologist (WB) to confirm the diagnosis of invasive breast cancer.

### Immunohistochemistry staining and fluorescence *in situ *hybridization analysis

All samples from primary tumor and from brain metastases were restained, and immunohistochemistry (IHC)-based expression for ERα, PR, and HER2 was determined in the central laboratory by a pathologist (WB) who was blinded to original assessments and to expression in the paired samples. In patients with more than one brain metastasis, only the single most representative lesion was subjected to receptor analysis.

Expression of ERα and PR was evaluated in the automated stainer (AutostainerLINK 48; DAKO, Glostrup, Denmark) with the use of clone SP1 (DAKO) and clone 636 (DAKO) antibodies, respectively. Signals were detected by EnVision FLEX kit. Expression of HRs was scored by using the semiquantitative Allred system, which takes into account the proportion of positive cells (graded 0 to 5) and staining intensity (graded 0 to 3) [23]. The proportion of positive cells and intensity were then summed to produce total scores of 0 or 2 through 8. A score of 0 or 2 was regarded as negative, whereas a score of 3 to 8, as positive. A positive result of either ERα or PR classified the case as HR-positive. In additional analyses, the currently recommended more-stringent criteria for HR positivity (≥ 1% staining) were used [24].

HER2 protein expression was determined by using semiquantitative IHC with HER-2/neu Test (clone 4B5; Ventana Medical Systems, Inc., Tucson, AZ, USA) and ultraVIEW Universal DAB detection kit (Ventana) in an automated stainer (Benchmark XT; Ventana). Samples with strong, complete, homogeneous membrane staining in > 30% of tumor cells (scored 3+) were considered positive, irrespective of the *HER2 *gene copy number determined with fluorescence *in situ *hybridization (FISH). The samples showing intermediate expression (scored 2+) were subjected to additional analysis of *HER2 *gene copy number by using FISH. FISH testing was also performed on brain metastatic lesions with conversion to HER2 3+ determined with IHC. Gene amplification with FISH was defined as a FISH ratio (*HER2*/centromeric probe for chromosome 17 ratio) of greater than or equal to 2.0. All FISH-positive patients were considered HER2-positive.

For all receptors, results were considered concordant if primary and metastatic tumor were both positive or both negative by using the previously mentioned criteria, whereas other combinations were considered discordant.

### Statistical analysis

The status of HRs and HER2 of metastatic brain tumors was compared with that of matched primary tumors and presented as discordance rate, its 95% confidence interval (CI), and the Cohen kappa statistics. The level of agreement based on *κ *values was assessed by using the Landis and Koch criteria: 0.00 to 0.20, slight agreement; 0.21 to 0.40, fair agreement; 0.41 to 0.60, moderate agreement; 0.61 to 0.80, substantial agreement; and 0.81 to 1.00, almost perfect agreement [25].

The proportions between paired groups were compared by using a χ^2 ^test or Fisher Exact test when appropriate. Overall survival, calculated from the date of breast cancer diagnosis to the date of death or last follow-up, was computed by using the Kaplan-Meier method. Survival curves were compared by using the log-rank test. Hazard ratios (HRs) with 95% confidence intervals (95% CIs) were computed by using Cox regression models. For all analyses, a statistical significance level of 0.05 was used, with no adjustment for multiple testing. All calculations were performed with SPSS 13.0 (SPSS Inc., Chicago, IL, USA).

## Results

The study group included 120 breast cancer patients treated in eight Polish institutions between 1996 and 2011 (Table 1). The median follow-up for the entire group was 97 months (range, 6 to 176 months). All patients underwent surgery for primary breast cancer and for brain metastases. The most frequent pathologic type was invasive ductal carcinoma (82%), and 47% of the tumors were grade 3. ERα, PR, and HER2 in primary tumors were positive in 42%, 34%, and 47% of patients, respectively. The analyses using ≥ 1% staining as a criterion of HR positivity showed virtually the same results and were not presented, as in the entire group, only one patient had to be reclassified (from ERα negativity to ERα positivity). In total, 29% of tumors were triple-negative. Almost all brain metastases (97.5%) were metachronous (that is, appearing > 2 months after the diagnosis of breast cancer had been rendered). In 60% of patients, brain was the first distant site of relapse. The vast majority of patients had either unifocal (61%) or two to three brain lesions (30%). Cerebellum was the most common site of brain metastases (27%), followed by parietal and frontal lobes (19% each). Most patients received chemotherapy, and more than 40% received endocrine therapy in the (neo)adjuvant or metastatic settings before brain surgery. Fifty-three percent of HER2-positive patients received trastuzumab in one of these settings.

**Table 1:** Patient characteristics^a^

Variable	Number	%
Breast cancer type		
Ductal	98	82
Lobular	10	8
Ductal/lobular	2	2
Uncertain	4	3
Other	5	4
Grade		
1	6	5
2	41	34
3	56	47
Unknown	17	14
ER		
Positive	51	42
Negative	69	57
PR		
Positive	40	34
Negative	78	65
Not determined	1	1
HER2		
Positive (IHC3^+^)	51	42
Positive (IHC2^+ ^and FISH^+^)	6	5
Negative	62	52
Not determined	1	1
Chemotherapy before brain metastases		
Yes, adjuvant, neoadjuvant	47	39
Yes, for metastatic disease	9	7
Yes, a combination thereof	47	39
No	8	7
Unknown	9	7
Endocrine therapy before brain metastases^b^		
Yes, adjuvant, neoadjuvant	31	26
Yes, for metastatic disease	5	4
Yes, a combination thereof	13	11
No	66	55
Unknown	5	4
Trastuzumab before brain metastases^c^		
Yes	30	53
No	25	43
Unknown	2	3
Chemotherapy after brain surgery		
Yes	57	47
No	47	39
Unknown	16	13
Endocrine therapy after brain surgery^b^		
Yes	21	17
No	86	72
Unknown	13	11
Anti-HER2 therapy after brain surgery^c^		
Trastuzumab	9	16
Lapatinib	7	12
Sequentially both	3	5
Type of first progression		
Local	2	2
Regional	9	7
Distant	106	88
Combined local regional and/or distant	1	1
Unknown	1	2
Dominant site of metastatic disease^d^		
Soft tissue	3	2
Bone	5	4
Visceral	110	92
Unknown	2	2
	
Age at breast cancer diagnosis (mean (range) years)	49 (26-80)	
Age at brain metastasis surgery (mean (range) years)	52 (29-83)	

^a^Percentages may not sum to 100 because of rounding to full numbers; ^b^tamoxifen, aromatase inhibitors, or tamoxifen and aromatase inhibitors; ^c^in HER2-positive patients (IHC3^+ ^or IHC2^+ ^and FISH^+^); ^d^category of worst prognosis irrespective of the extent, in order: soft tissue, bone, visceral; see Materials and methods. Number of patients, 120. ER, estrogen receptor; HER2, epidermal growth factor receptor; PR, progesterone receptor; WBRT, whole-brain radiotherapy.

**Figure 1:**
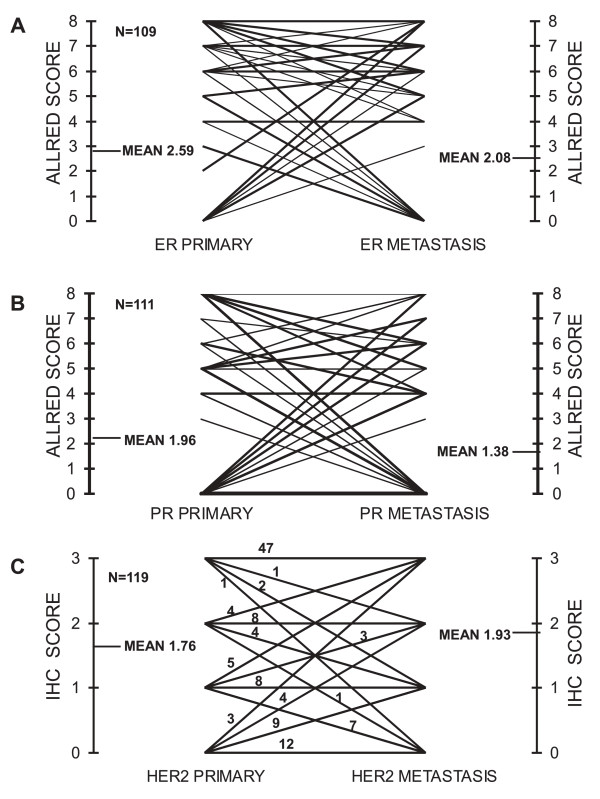
**Phenotypes for ER (A), PR (B), and HER2 (C) in primary tumors and their matched brain metastases**. ER, estrogen receptor; PR, progesterone receptor; HER2, epidermal growth factor receptor 2.

Conversion of HR status mostly included loss of receptor expression in brain metastases by originally positive breast carcinoma (Table 2). Conversion of ERα status occurred in 35 (29% (95% CI, 21% to 38%); *κ *= 0.389) of 120 patients, including 22 (43%) of 51 patients who lost original expression in metastatic foci and 13 (19%) of 69 patients who acquired it (*P *= 0.005) (Figure 1). PR status changed in 34 (29% (21% to 38%); *κ *= 0.320) of 119 patients; in 23 (56%) of 41 patients from positive to negative, and in 11 (14%) of 78 patients from negative to positive (*P *< 0.001). The subsets of tumors with ERα and PR conversions overlapped partially (22 (63%) of 35 tumors with ERα conversion showed also PR conversion).

**Table 2:** Paired analysis of receptor expression in breast cancer and brain metastases

		Brain metastasis		
			
Primary tumor		Negative	Positive	Total number (%)
ER	Negative, *n *(%)	56 (81)	13 (19)	69 (100)
	Positive, *n *(%)	22 (43)	29 (57)	51 (100)
	Total, *n *(%)	78 (65)	42 (35)	120 (100)
		
	Overall discordance rate (95% CI)	29% (21% to 38%)		

PR	Negative, *n *(%)	67 (85)	11 (15)	78 (100)
	Positive, *n *(%)	23 (56)	18 (44)	41 (100)
	Total, *n *(%)	90 (76)	29 (24)	119 (100)
		
	Overall discordance rate (95% CI)	29% (21% to 38%)		

HER2	Negative, *n *(%)	51 (84)	10 (16)	61 (100)
	Positive, *n *(%)	7 (12)	51 (88)	58 (100)
	Total, *n *(%)	58 (49)	61 (53)	119 (100)
		
	Overall discordance rate (95% CI)	14% (9% to 22%)		

Conversion of HER2, considering IHC and FISH, occurred in 17 (14%; 95% CI, 9% to 23%]; *κ *= 0.464) of 119 patients; in seven (12%) of 58 patients from positive to negative, and in 10 (16%) of 61 patients from negative to positive (*P *= 0.60). Interestingly, among nine patients with IHC HER2 conversion to 3+ who were assessable with FISH, only three proved to have underlying *HER2 *gene amplification. The number of cases with FISH measurement in the primary tumor and in the corresponding brain metastases was too small for meaningful analysis.

No relation was found between the changes of HRs and HER2 (*P *= 1.0).

Because more than 80% of patients received chemotherapy before brain metastases, its impact on conversion cannot be reliably assessed. Trastuzumab did not significantly affect conversion of HER2 (change in two of 31 patients versus four of 28 patients not administered trastuzumab; *P *= 0.22), whereas endocrine therapy strongly influenced conversion of both HRs (ER, 20 of 49 and 13 of 66 in patients who did and did not receive endocrine therapy, respectively; *P = *0.021; PR, 22 of 48 and 10 of 66, respectively; 0.001). Most ER and PR conversions after endocrine therapy were toward the loss of both HRs (85% and 82% of all conversions, respectively).

Finally, the conversion rate of any receptor did not associate with time from primary diagnosis to brain surgery considered as a continuous variable (*P *= 0.64, 0.91, and 0.87, for ERα, PR, and HER2, respectively).

The median overall survival from primary diagnosis in the entire group was 4.7 years (95% CI, 3.3 to 6.1), and 5-year survival was 48% (95% CI, 38% to 57%).

**Figure 2:**
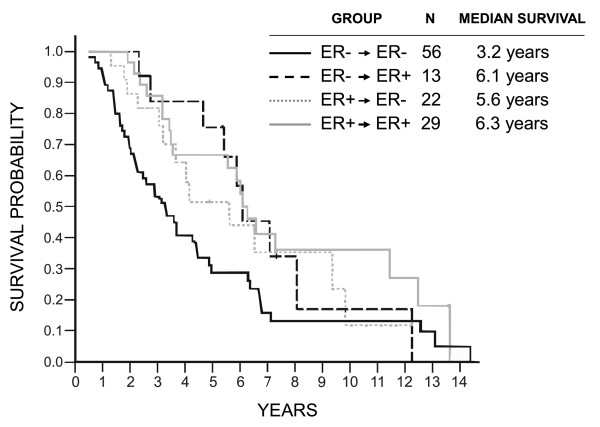
**Overall survival in relation to ERα conversions**. Log-rank *P *= 0.041 (all comparisons); *P *= 0.005 (ER^-^/ER^- ^versus others); HR = 1.84 (1.20 to 2.83; ER^-^/ER^- ^versus others).

**Figure 3:**
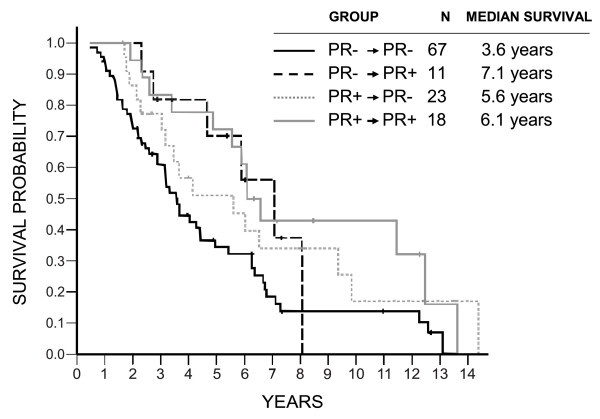
**Overall survival in relation to progesterone receptor (PR) conversions**. Log-rank *P *= 0.037 (all comparisons); *P *= 0.005 (PR^-^/PR^- ^versus others); HR = 1.89 (1.22 to 2.94; PR^-^/PR^- ^versus others).

**Figure 4:**
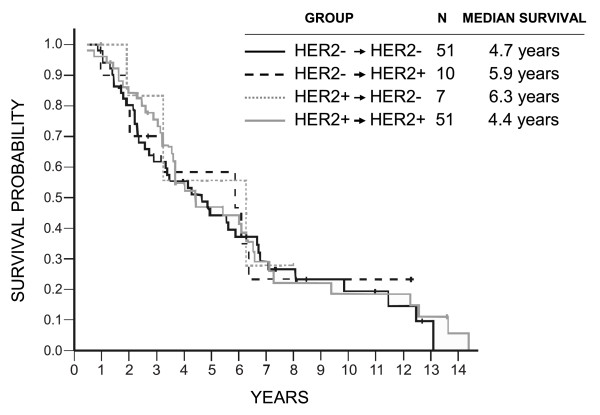
**Overall survival in relation to epidermal growth factor receptor 2 (HER2) conversions**. Log-rank *P *= 0.944 (all comparisons); *P *= 0.582 (PR^-^/PR^- ^versus others); HR = 1.13 (0.73 to 1.74; HER2^-^/HER2^- ^versus others).

Patients who were ERα or PR negative in the primary tumors and retained their negativity in their brain metastases did significantly worse than the other patients (ERα, log-rank *P = *0.005; HR = 1.84 (95% CI, 1.20 to 2.83]; PR, *P = *0.005; HR, 1.89 (95% CI, 1.22 to 2.94)), but no apparent impact of ERα and PR conversion was found either way on survival (Figures 2 and 3). Similarly, no prognostic impact of HER2 conversion was found (Figure 4).

After the brain surgery (missing data excluded), 80% of patients received whole-brain radiotherapy, 55%, chemotherapy, 20%, endocrine therapy, and 25% of HER2-positive patients based on primary tumor assessment received anti-HER2 therapy (trastuzumab, lapatinib, or sequentially both), including three patients who received lapatinib based on HER2 gain in the brain lesion. In some patients, receptor status in brain metastases was not determined right after the excision (exact number unknown), so no attempt was made to evaluate the impact of conversion on subsequent systemic therapy.

## Discussion

We performed comprehensive analyses of ERα, PR, and HER2 expression in primary tumors and in paired brain metastases in a large series of breast cancer patients. As expected, two phenotypes of primary tumor, HER2-positive and triple negative, were clearly overrepresented in this series (47% and 29%, respectively), as both are associated with a particularly high risk of brain relapse [1-4]. Additionally, almost half of primary tumors were poorly differentiated, and only a few low-grade tumors were found.

Our study demonstrated a relatively high rate of HR conversion in brain metastases from breast cancer. A switch of ERα and PR (29% each) was within the wide range of conversions reported earlier for other sites of relapse [8-11,26,27].

HR conversion in the brain has not been studied extensively, as in most studies, the brain constituted only a small fraction of all metastatic sites. A few small studies (23 to 44 patients) specifically addressing brain metastases showed a broad range (6% to 44%) of HR conversion [4,17-21]. None of these studies was large enough for meaningful analysis of factors influencing conversion and related prognosis.

Similar to that of other metastatic sites [9,10,12,28], conversion of ERα and PR in brain metastases in our series resulted mainly in loss of ERα and PR (63% and 68% of all conversions, respectively). This phenomenon is likely a result of tumor heterogeneity and the development of brain metastases from a minority of cells present within the primary tumor, as shown in a recent study using next-generation sequencing technologies [29]. Less-likely causes include clonal selection of undifferentiated HR-negative tumor cells from an original heterogeneous population of tumor cells during endocrine therapy, or silencing of HR expression due to activation of growth-factor signaling [30,31].

Some studies have suggested that HR discordance, in particular toward loss of HRs in metastatic sites, is related to shorter survival [12,13,32], which might have been due to inappropriate therapy or to selection of treatment-resistant tumor clones with unstable and more aggressive phenotypes. We were unable to confirm this finding; however, relatively few patients presented with HR-positive primary tumor, and our analysis was thus underpowered. A few patients in our series demonstrated gain of HRs, a finding that may support a repeated measurement, preferably including retesting of primary tumors.

In our study, HER2 status in brain metastases was less prone to changes compared with HRs, and conversions in both directions were more balanced. The overall conversion rate of 14% is close to a pooled HER2 discordance rate of 11.5% for distant metastases at various locations demonstrated in a recent systematic review of 26 studies [14]. We did not find any clinical factors influencing HER2 conversion. In the previously mentioned review, metachronous (versus synchronous) occurrence of metastases was the only factor increasing the HER2 conversion rate. A recent study including 182 patients demonstrated a conversion rate of HER2 in 24% of metastases, and this phenomenon was correlated with preceding chemotherapy and not with trastuzumab therapy [33].

HER2 conversion in brain metastases was reported in a few studies, and the results seem to be similar compared with other metastatic sites [4,8-22]. However, all these studies were relatively small (up to 44 patients) [9]; thus any comparisons should be interpreted with caution. Interestingly, one small study suggested a full concordance of HER2 status in brain metastases determined with FISH, as opposed to IHC assessment [22]. Nevertheless, we verified FISH status in all patients with positive HER2 IHC expression in the brain lesions, and indeed, the proportion of FISH-positive cases among otherwise IHC HER2-positive cases seemed to be lower compared with respective figures in primary tumors. This finding may be related to heterogeneity of primary tumors or to specific features of the brain microenvironment [34,35]. The question of biologic behavior of HER2 IHC-positive, FISH-negative metastatic brain tumors and particularly their responsiveness to anti-HER2 therapy remains to be established. Similar to that of HRs, conversion of HER2 did not apparently influence survival, and no clinical factors were associated with this phenomenon.

Discordant phenotypes in primary and recurrent breast cancers have usually been interpreted as a genuine switch in tumor biology or intratumoral heterogeneity, with treatment-related selection of more-aggressive and resistant clones [36,37]. An apparent loss of HRs in patients administered endocrine therapy in this series seems to support strongly the latter hypothesis. However, some differences may also be due to limited accuracy and reproducibility of receptor assays and to sampling errors related to focal intratumor heterogeneity, and the relative contributions of all these factors are unknown [38]. Notably, a study using gene expression and genomic hybridization techniques suggests relative genomic stability of breast cancer over time [39], although this may not apply to brain metastases [40].

Possible differences in preanalytic procedures (such as tissue handling before fixation, quality and length of fixation), immunostaining and interpretation of results are particularly important in retrospective multiinstitutional studies on tissue tumor markers. In our study, the assessments were made centrally on newly prepared immunostained sections and in a blinded manner, minimizing the risk of analytic discordance and interobserver variability. By using full tissue sections, we also decreased the impact of intratumoral heterogeneity and problems with the reliability of biomarker expression in small biopsy specimens [41]. However, our results should still be interpreted with caution, as we were unable to control for preanalytic procedures and to assess their impact.

The most important question related to changing tumor phenotypes in metastatic lesions is the clinical relevance of these events. A relatively high discordance rate may suggest the clinical utility of repeated immunostaining of recurrent lesions, because currently used systemic therapies of advanced breast cancer are widely dependent on the appropriate targeting of HRs and HER2. At least three prospective studies demonstrated a change of clinical management in 14% to 20% of breast cancer patients as a result of discordance between primary and metastatic tumors [26-28]. The reevaluation of metastatic breast cancer lesions is now recommended by the European Society of Medical Oncology and the American Society of Clinical Oncology [42,43]; however, no particular recommendations were made in relation to brain metastases. Specific features of brain metastases include their low accessibility for a biopsy and a limited blood-brain barrier penetrance for most cytotoxic agents and trastuzumab. The array of effective systemic therapies in breast cancer brain metastases may, however, soon be expanded by emerging targeted agents. For example, a recent phase II study demonstrated an impressive volumetric response rate of 67% (29 of 43 patients) with a combination of lapatinib and capecitabine in radiotherapy-naïve brain metastases in HER2-positive breast cancer [44].

In our series, in all cases, brain metastasis samples were obtained at surgical excision, allowing subsequent analysis. Nevertheless, in some patients, immediate receptor measurement in brain lesions was not performed locally, and its impact on clinical decisions could not be reliably assessed. The results of our study cannot also be extrapolated to inoperable brain metastases, and the relevance of biopsy attempts in such cases remains unknown. Rationally, this decision should be based mainly on clinical judgment, and a biopsy considered, if its results would affect clinical decision making, or if a possibility exists of unrelated disease (second primary tumor or benign lesion). However, the biopsy attempt should be balanced, owing to technical difficulties of obtaining accessible tissue from the brain lesion and to patient reluctance. In the case of discordant results, a retesting of primary tumor by using the same methods should be considered.

## Conclusions

Our study demonstrates that, in a proportion of breast cancer patients, the receptor phenotype of brain metastases does not reflect its status in primary tumor. This might suggest that missing this discordance, particularly in the case of receptor gain, may deny some patients a potentially effective systemic therapy. However, a verification of this assumption in a prospective clinical trial including a random patient assignment to therapy based on the assessment of metastatic versus primary lesions does not seem feasible. Nevertheless, available data suggest that in routine clinical practice, repeated immunostaining of excised brain metastases seems to be worthwhile. The biopsy of an inoperable brain lesion should be weighed against technical difficulties and the risk of such procedure, and should always be a joint decision between the patient and physician.

## Abbreviations

CI: confidence interval; ERα: estrogen receptor alpha; FISH: fluorescence *in situ *hybridization; HER2: epidermal growth factor receptor 2; IHC: immunohistochemistry; PR: progesterone receptor.

## Competing interests

The authors declare no conflicts of interest.

## Authors' contributions

RD was responsible for study conception and design, coordination, data analysis and interpretation, and manuscript writing; RDZ, for study design, statistical analysis and interpretation, and manuscript writing; TT, TM, WO, BCA, BR, WO, FS, BJ, and AK, for provision of study materials and patients, collection and assembly of data, and data interpretation; JL, for molecular analysis and data analysis and interpretation; WB, for pathological review, and data analysis and interpretation; and JJ, for study conception and design, coordination, data analysis and interpretation, and manuscript writing. All authors read and approved the final manuscript.

## Authors' information

Renata Duchnowska, MD, PhD, oncologist; Rafał Dziadziuszko, MD, PhD, oncologist; Tomasz Trojanowski, MD, PhD, neurosurgeon; Tomasz Mandat, MD, PhD, neurosurgeon, Waldemar Och, MD, neurosurgeon; Bogumiła Czartoryska-Arłukowicz, MD, oncologist; Barbara Radecka, MD, PhD, oncologist; Wojciech Olszewski, MD, PhD pathologist; Franciszek Szubstarski, MD, PhD, pathologist; Wojciech Kozłowski, MD, PhD, pathologist; Bożena Jarosz, MD, PhD, pathologist; Wojciech Rogowski, MD, PhD, oncologist; Anna Kowalczyk, MD, PhD, oncologist; Janusz Limon, MD, PhD, biologist; Wojciech Biernat, MD, PhD, pathologist; Jacek Jassem, MD, PhD, oncologist^.^
